# Hijacking and Use of Host Kinases by Chlamydiae

**DOI:** 10.3390/pathogens9121034

**Published:** 2020-12-10

**Authors:** Prakash Sah, Erika I. Lutter

**Affiliations:** Department of Microbiology and Molecular Genetics, Oklahoma State University, Stillwater, OK 74078, USA; prakash.sah@okstate.edu

**Keywords:** *Chlamydia*, kinase, infection, phosphorylation, signaling

## Abstract

*Chlamydia* species are causative agents of sexually transmitted infections, blinding trachoma, and animal infections with zoonotic potential. Being an obligate intracellular pathogen, *Chlamydia* relies on the host cell for its survival and development, subverting various host cell processes throughout the infection cycle. A key subset of host proteins utilized by *Chlamydia* include an assortment of host kinase signaling networks which are vital for many chlamydial processes including entry, nutrient acquisition, and suppression of host cell apoptosis. In this review, we summarize the recent advancements in our understanding of host kinase subversion by *Chlamydia*.

## 1. Introduction

Chlamydiae species are obligate intracellular pathogens that represent a significant burden to healthcare and the economy. Human infections are caused by two species: *Chlamydia trachomatis* and *Chlamydia* (*Chlamydophila*) *pneumoniae*. *C. trachomatis* causes infections of ocular and genital tract epithelium [1]. Ocular infections caused by *C. trachomatis* are the leading infectious cause of preventable blindness worldwide [2]. Genital tract biovars comprised of serovars D-K are the most frequently reported bacterial sexually transmitted infections (STIs) in the United States [3]. The majority of infections are asymptomatic which facilitate transmission of this pathogen. Chronic infections lead to complications such as pelvic inflammatory disease (PID), ectopic pregnancy, and infertility. Furthermore, *C. trachomatis* infections can facilitate HIV transmission and have been associated with an increased risk of cervical cancer [4,5]. Lymphogranuloma venereum (LGV) biovars comprised of serovars L1-L3 cause invasive infections of the urogenital tract [5]. *C. pneumoniae* is a frequent cause of community acquired pneumonia and has been associated with several chronic diseases such as atherosclerosis [6,7]. Non-human infections are caused by various Chlamydiae strains including *Chlamydia muridarum, Chlamydia caviae* and *Chlamydia psittaci. C. muridarum* is a mouse adapted strain used as model for studying genital infections while *C. caviae* and *C. psittaci* are pathogens of veterinary importance [8,9]. *C. psittaci* is a known zoonotic agent causing respiratory infections in humans and recently *C. caviae* is also emerging as a zoonotic agent [10,11,12].

Despite variations in infections caused and hosts targeted, *Chlamydia* species all share a common infection cycle. *Chlamydia* have a unique biphasic developmental cycle consisting of alternation between two distinct forms: the infectious elementary bodies (EBs) and the non-infectious reticulate bodies (RBs) [13,14]. Upon contact with the host cell, EBs mediate uptake by the host in a membrane bound compartment called an inclusion and avoids fusion with lysosomes [15]. EBs transition to RBs within the inclusion and undergo several rounds of replication. About mid-way through the life cycle RBs asynchronously start to re-differentiate back into EBs. At the end of the life cycle, about 48–72 h, EBs are released from the host cell via lysis or extrusion to infect neighboring cells [16]. 

Being obligate intracellular in nature, *Chlamydia* are reliant on various host cell process for their growth and development. From within the inclusion, *Chlamydia* usurps various host processes to acquire nutrients, modulate host immune response, maintain inclusion integrity, and prevent apoptosis until exit. Many of these host cell processes are regulated via phosphorylation events by several host kinases. Thus, subversion of the host kinases may be integral to the intracellular life style of *Chlamydia.* Several studies have highlighted the roles of host kinases in different stages of *Chlamydia* life cycle to promote growth and development. Within this review, we focus on the various roles that host kinases play in different aspects of chlamydial infections. A summary of host kinases subverted by *Chlamydia* is shown in Table 1. 

## 2. Host Kinases Important for Chlamydial Entry and Invasion

The very first step in the infection cycle is invasion and entry, a process that utilizes many host kinases. *Chlamydia* employs several adhesins that bind to receptors on the host cells to promote attachment and entry into the host cell. Among these, several receptor tyrosine kinases (RTKs), including platelet derived growth factor receptor (PDGFR), fibroblast growth factor receptor (FGFR), and EphrinA2 receptor (EphA2) are utilized for chlamydial attachment and subsequent host cell entry [18,31,34]. Overall, RTKs play a similar role in chlamydial infection in that they contribute to binding/invasion and activating downstream signaling to regulate other aspects of chlamydial development. Utilizing a large scale high throughput RNA interference screen identified PDGFRβ as important host factor for *C. muridarum* binding and invasion [18]. PDGFRβ was suggested as a receptor for *C. muridarum* binding as siRNA mediated depletion of PDGFRβ or treatment with neutralizing antibody against PDGFRβ resulted in a significant decrease in binding to host cell [18]. The same study also found that independent activation of Abl kinase was required for invasion. Activation of PDGFRβ and/or Abl kinase led to phosphorylation of actin modulating proteins Vav2, WAVE2, and cortactin [18]. Given its role in binding and invasion, PDGFRβ has been identified as potential therapeutic target for topical siRNA based therapy. Recently, PDGFRβ knockdown via siRNA-encapsulated nanoparticles has been shown to decrease *C. trachomatis* infection [59]. *C. trachomatis* and *C. muridarum* EBs also bind to fibroblast growth factor 2 (FGF2) enabling their attachment to FGFR leading to localized FGFR activation and entry of EBs into non-phagocytic cells [31]. Further, *C. trachomatis* and *C. muridarum* stimulate FGF2 expression via extracellular signal-regulated kinase ½ activation (ERK1/2) and its release from the host cell facilitating subsequent rounds of infection [31]. Inhibition of both FGFR and PDGFR using chemical inhibitors resulted in a significant decrease in internalization of *C. muridarum* while only a small effect was seen when either FGFR or PDGFR was inhibited indicating these two RTKs may function redundantly in Chlamydial invasion [31]. However, the bacterial factors that mediate FGF2 and PDGFR binding are currently unknown. In *C. trachomatis* infections, there are distinct functional requirements of EGFR and PDGFR. Specifically, EGFR activation was found to be important for attachment, internalization, inclusion development, and F-actin assembly around the inclusion while PDGFR was only required during initial chlamydial attachment [18,33]. An RNA interference screen identified EphA2 as another receptor for *C. trachomatis* entry which was confirmed via adherence assay [34]. EphA2 was shown to be a chlamydial adherence and invasion protein required for *C. trachomatis* development and inhibiting apoptosis by activating the Phosphatidylinositol-3 kinase (PI3K) pathway [34]. Multiple studies have shown that inactivation of PI3K, Rac or Cdc42 inhibit host cell invasion, however, the mechanism of PI3K activation is yet to be deciphered [60,61,62]. During replication, activated EphA2 accumulates around the inclusion interacting with PI3K (at the p85 regulatory subunit) thereby contributing to the activation of the PI3K/Akt signaling pathway which is subsequently essential for normal chlamydial development [34]. Akt phosphorylation at Ser 473 occurs in a binomial manner, early in infection and again around 12 h post-infection [38].

Focal adhesion kinase (FAK) recruitment is important for *C. caviae* invasion as invasion efficiency of *C. caviae* is markedly reduced in FAK- and vinculin-knockout cells [24,63]. FAK recruitment is mediated by the translocated actin recruitment protein (TarP) [24]. TarP contains a leucine-aspartate (LD) motif in the F-actin binding domain (FAB1) that binds FAK and mediates actin assembly in a Cdc42 and Arp2/3 dependent manner [24,64]. Unlike TarP from other chlamydial species, the *C. pneumonaie* ortholog lacks the binding domain for FAK [24,64]. However, *C. pneumoniae* can alternatively activate FAK signaling in addition to MAP kinase kinase (MEK)/extracellular signal-regulated kinase (ERK) and PI3K for entry and invasion [23]. A subsequent study showed that *C. pneumoniae* polymorphic membrane protein 21 (pmp21) binds to epidermal growth factor receptor (EGFR). siRNA depletion of EGFR significantly reduced *C. pneumoniae* adhesion and internalization while ectopic expression of EGFR in receptor-negative cells resulted in increased adhesion and internalization [32]. *C. pneumoniae* EBs binding to the EGFR receptor activated the MAP kinase pathway resulting in rapid activation and phosphorylation of ERK1/2[32]. Overall, chlamydial host cell entry is a complex process that is highly reliant on host kinases and signaling networks for remodeling cytoskeletal actin.

## 3. Host Kinases in Nutrient Acquisition by *Chlamydia*

*Chlamydia* have a reduced genome, lacking genes for proteins in several metabolic pathways and thus are dependent on the host for its many metabolic requirements [65]. Host derived lipids are important for bacterial replication, inclusion development, homotypic fusion of inclusions, conversion of RBs to EBs, and reactivation from persistence [47,66,67]. Host glycerolipids are acquired and modified by *C. trachomatis*, a process that requires cytosolic phospholipase A2 activation via the MAP kinase pathway (Ras/Raf/MEK/ERK) [48,68].

Rottlerin, an inhibitor of several host serine/threonine kinases including protein kinase C δ (PKCδ) was shown to affect *C. trachomatis* development and block sphingolipid acquisition [46]. A previous study had shown PKCδ to be in proximity of the inclusion [45]. Based on this, it was suggested that PKCδ may play a role in lipid acquisition. However, since then rottlerin has been discredited as specific inhibitor of PKCδ [69] and hence the role of PKCδ in lipid acquisition needs further investigation. Most recently, multiple PKC isoforms were found to be localized to inclusion microdomains in *C. trachomatis* [44]. Additional roles for PKC are discussed later regarding inhibition of apoptosis.

Phosphatidylinositol 4-kinase II-alpha (PI4KIIα), a host kinase that generates Phosphatidylinositol-4-phoshate (PI4P), is recruited to the inclusion. While PIP4 is important for infectivity as seen by siRNA depletion [35], it is not necessary for CERT recruitment to the inclusion [70,71], thereby leaving its role at the inclusion undefined. It has, however, been suggested that the abundance of PI4P at the inclusion may recruit PI4P binding proteins that regulate lipid trafficking, but this has yet to be determined [47].

A study using siRNA depletion screens identified Fyn, a Src family kinase (SFK) member that also localizes at microdomains, to be important for sphingolipid acquisition by *C. trachomatis* [26]. SFKs at microdomains regulate inclusion trafficking along microtubules to the microtubule organization center (MTOC) and hence may hijack the vesicular transport along microtubules. A recent study found *C. trachomatis* utilizes the Akt/AS160 signaling pathway to acquire sphingolipids via Rab14 mediated vesicular transport [41]. Akt remains phosphorylated and recruited to the inclusion membrane for the duration of infection and is essential for chlamydial replication, inclusion size, and infectivity [41].

## 4. Src Family Kinase Rich Microdomains

Active SFKs, non-receptor membrane associated tyrosine kinases, co-localize with at least nine inclusion membrane proteins (Incs) including IncB, IncC, CT101/Myosin Regulatory Complex subunit A (MrcA), CT222, CT223, CT224, CT228, CT288, and CT850 at discrete cholesterol rich sites called microdomains on *C. trachomatis* inclusions [27,28,72]. Interestingly, SFK recruitment, specifically Src and Fyn, is only observed in human *Chlamydia* infecting species (*C. trachomatis* and *C. pneumoniae*) [27]. These microdomains on the chlamydial inclusion have been shown to be a platform for regulating various processes important for *C. trachomatis* development. Inhibition of Src kinase or growth in a Src deficient cell line resulted in significant reduction in association of *C. trachomatis* inclusions with the MTOC as well as reduced infectious progeny production [25]. Thus, SFKs regulate microtubule dependent trafficking of the nascent inclusion to the MTOC and intracellular development [25]. *C. caviae* and *C. muridarum* do not recruit SFKs and do not traffic to the MTOC [27]. In addition, chemical inhibition of Src or growth in a Src deficient cell line led to an increase in infectious progeny production suggesting that Src may play a role in restricting growth of these *Chlamydia* species [25].

**Figure 1:**
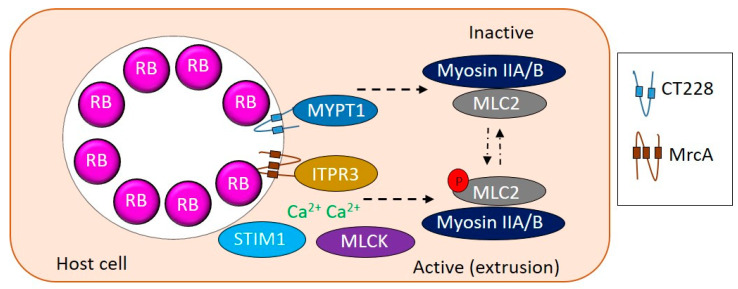
Modulation of extrusion by *Chlamydia*. CT228 and MrcA interact with MYPT1 and ITPR3, respectively, to regulate the phosphorylation state of MLC2. Active phosphorylated MLC2 correlates with extrusion whereas inactive unphosphorylated MLC2 correlates with lysis. ITPR3 along with STIM1 control Ca2+ concentrations to influence relative active levels of MLCK and MYPT1 to regulate extrusion.

CT228 and CT101/MrcA have been shown to regulate extrusion via the myosin phosphatase pathway (Figure 1) [28,29,30]. CT228 binds to and recruits MYPT1 to the chlamydial inclusion [28,30]. MYPT1 phosphorylated at residues T853 and T696 colocalizes with active SFK laden microdomains along with other proteins of myosin pathway, Myosin light chain 2 (MLC2), Myosin light chain kinase (MLCK), and the heavy chains, Myosin IIA and B [28]. siRNA mediated depletion of MLCK, MLC2, Myosin IIA and B resulted in decreased extrusion production by *C. trachomatis* [28]. MrcA binds to the inositol 1,4,5-triphosphate receptor 3 (ITPR3) which works with stromal interaction molecule 1 (STIM1) to regulate Ca^2+^ release, ensuring MLCK is active and phosphorylates MLC2 to regulate extrusion [28,29]. Overall, a complex interplay between MLCK and a phosphatase at the SFK microdomains regulates chlamydial exit strategies and determines whether *C. trachomatis* exits the host via lysis or extrusion.

## 5. Phosphorylation of *Chlamydia* Effectors by Host Kinases

*C. trachomatis* encodes a T3SS to secrete effector proteins that localize into the host cytosol or inclusion membrane [65,73]. The well-known TSSS effector, TarP, is required for actin recruitment during *Chlamydia* entry into the host cell [17]. TarP modulates actin via activity of different domains that resemble mammalian signaling motifs, recruiting actin and Arp2/3 complex [74]. The tyrosine-phosphodomain in the N-terminal of TarP is only found in *C. trachomatis* serovars and is phosphorylated by host kinases (Src, Abl, Syk) leading to recruitment of the Arp2/3 complex [17,18,19,20,39,60,75]. TarP phosphorylation is not necessary for F-actin assembly as TarP can activate FAK directly by mimicking the FAK cofactor paxillin [24,64]. This is due to the C-terminal being highly conserved among *Chlamydia*, which contains an LD motif that binds to FAK leading to recruitment of Arp2/3, except in *C. pneumoniae* which lacks a LD motif [24,64].

Translocated early phospho-protein (TepP) is another T3SS secreted effector that is tyrosine phosphorylated by the host kinase Src at multiple tyrosine residues after delivery into the host cell [21,22]. TepP recruits Crk adaptor proteins, GSK3β, and PI3K to the early inclusion, independent of Src phosphorylation and plays a role in regulating the innate immune response to *Chlamydia* [21]. Activated PI3K signaling leads to phosphorylation of phosphoinositide (4,5)-bisphosphate (PIP2) to generate phosphoinositide (3,4,5)-triphosphate (PIP3) which is localized on early inclusions [21]. Interestingly, PI3K activation is spatially limited to the inclusion and does not include the PI3K at the plasma membrane, and as such Akt is not activated [21]. Overall, these studies demonstrate that TepP can modulate PI3K and may have a role in membrane trafficking.

Incs are chlamydial effectors that are translocated across and inserted into the inclusion membrane facing the host cytosol [76,77,78,79]. IncA was the first Inc effector to be discovered in *C. psittaci* and was reported to be phosphorylated by host kinases [80,81]. *C. trachomatis* IncG was subsequently shown to be phosphorylated in the host cell as well [82]. While the role of IncA/IncG phosphorylation in chlamydial biology/pathogenesis is unknown, recent studies have indicated the possibility of more Incs being phosphorylated by host kinases. A large-scale Human–Inc interactome study found Incs binding to host kinases while proteome analysis of isolated mid-cycle *C. trachomatis* inclusions also showed association of various host kinases with the inclusion [83,84]. More recently, phosphoproteomic analysis of *C. trachomatis* infected cells revealed several chlamydial proteins, including several Incs, to be phosphorylated. Predicted host kinases that could phosphorylate chlamydial proteins included protein kinase A (PKA), PKC, casein kinase 2 (CK2), and GSK3β among other host kinases [54]. Interestingly, PKC and GSK3β have been shown to localize at the inclusion during infection [44,45]. Studying the role of Inc phosphorylation awaits future investigations. Such studies would require mutation analysis of phosphorylation sites on Incs. Recent advancements in chlamydial mutagenesis and availability of shuttle vectors with different selection markers will aid in these investigations. However, challenges such as low transformation efficiency and the time-consuming, laborious nature of chlamydial mutagenesis remain.

## 6. Apoptosis Resistance

**Figure 2:**
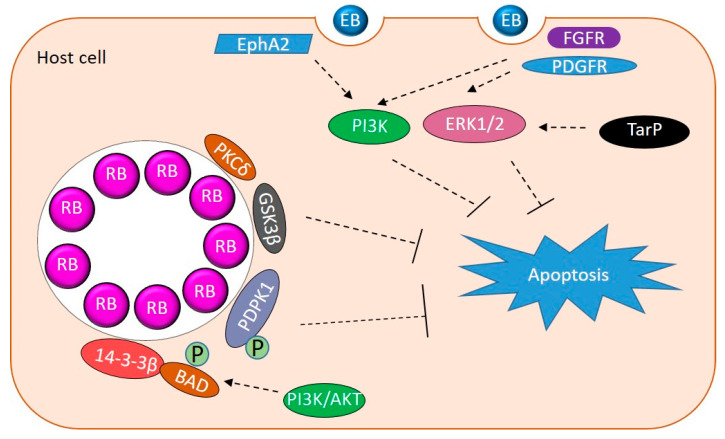
Host kinases manipulated by *Chlamydia* to promote survival. Schematic contains a summary of host kinases manipulated by *Chlamydia* to inhibit apoptosis. EB binding and entry activate PI3K and ERK1/2 to promote survival. TarP activates Erk1/2. Sequestration of PKC, 14-3-3β, PDPK1, and GSK3β work via different mechanisms to prevent apoptosis.

As an intracellular pathogen, it is important for *Chlamydia* to keep the host cell alive for optimal growth. *Chlamydia* promote host cell survival via activating pro-survival signaling, downregulating pro-apoptotic proteins, upregulating or stabilizing pro-survival proteins, and sequestering pro-apoptotic proteins to the inclusion (Figure 2) [85,86]. MEK/ERK signaling and PI3K pathways modulate apoptosis and survival and are activated during entry of *C. trachomatis*, *C. muridarum* and *C. pneumoniae* by their binding to host RTKs (discussed above) [18,31,32,34]. Phosphorylated TarP interacts with SRC homology 2 domain-containing transforming protein C1 (SHC1) during entry to promote host cell survival during *C. trachomatis* infection by activating MEK/ERK signaling [87]. It was observed that apoptosis resistance in *C. trachomatis* infected cells was mediated by ERK signaling and up regulation of the anti-apoptotic BCL-2 family member MCL-1 (followed by PI3K-dependent stabilization) and BAG family molecular chaperone regulator 1 (BAG1) [36,53,58]. During *C. trachomatis* infection, the PI3K pathway activates Akt. This PI3K/Akt activation contributes to maintaining pro-apoptotic BAD in a phosphorylated state which is sequestered by 14-3-3β at the inclusion [38]. 14-3-3β is released if PI3K is inhibited by LY294002 while depletion of AKT through short-interfering RNA reverses apoptosis resistance in *C. trachomatis* infected cells [38]. Downstream of PI3K signaling, 3-phosphoinositide-dependent protein kinase 1 (PDPK1) promotes apoptosis resistance in *C. trachomatis* infected cells by stabilizing MYC through phosphorylation at Ser 62. MYC stabilization leads to upregulation of hexokinase II (HKII) and enrichment of HKII at the mitochondria inhibiting cell apoptosis [42].

PKCδ sequestration to the *C. trachomatis* inclusion has been implicated to contribute to apoptosis resistance [45]. Recruiting PKCδ to the inclusion causes redistribution of this pro-apoptotic kinase away from the mitochondria and nuclease resulting in anti-apoptotic effects. Using co-immunoprecipitation and GST pull-down assays, *C. pneumoniae* Inc Cpn1027 has been shown to interact with members of the WNT-pathway, cytoplasmic activation/proliferation-associated protein-2 (CAPRIN2) and GSK3β. This interaction may contribute to apoptosis resistance by activating the transcription of pro-survival genes [43]. Interestingly, inhibition of WNT signaling via small molecule inhibitor, IWP2, has also been shown to impair *C. trachomatis* infection [88]. The T3SS effector TepP recruits GSK3β to the early *C. trachomatis* inclusion [21], however whether this interaction modulates WNT signaling remains unknown. Overall, interaction with these signaling pathways ensures that *Chlamydia*’s requirements are met without triggering host apoptosis.

## 7. Immunopathology of Chlamydial Infections and Association with Chronic Diseases

Chronic infection with *Chlamydia* has been epidemiologically linked with development of inflammatory diseases. *C. trachomatis* infection can lead to sequelae such as PID, infertility, and has been associated with development of cervical cancer [4,5]. Recognition of *Chlamydia* by the host cell results in an inflammatory response which is required for bacterial clearance but is also responsible for the immunopathology of chlamydial infections [85,86,89]. *C. trachomatis* induces proinflammatory interleukin-8 (IL-8) during infection via activation of ERK and p38 kinase signaling [49,50]. Contrary to this, ERK and p38 were also shown to upregulate production of the anti-inflammatory cytokine IL-10, which in turn downregulates production of pro-inflammatory cytokines via JAK/STAT3 signaling [52]. It has been suggested that ERK and p38 signaling may play a role maintaining a balance between pro- and anti-inflammatory response. Additionally, TepP recruits PI3K at the early inclusion to dampen the transcription of type 1 interferon induced genes [21]. The events downstream of PI3K activation which lead to the altered immune response are not yet known.

*C. trachomatis* promotes host DNA damage by eliciting reactive oxygen species production while impairing the DNA damage responses (DDR). Even with impaired DDR, *C. trachomatis* keeps its host proliferating via ERK signaling (among other signaling pathways) creating an environment that may predispose the host cell to malignant transformation [51]. *C. trachomatis* has also been shown to reduce the levels of tumor suppressor protein p53 via PI3K/AKT signaling activation during infection to overcome DNA-damage-driven cytotoxic response [37]. *C. trachomatis* induced epithelial-mesenchymal transition (EMT) has been implicated in molecular pathogenesis of sequelae associated *C. trachomatis* infections and its role as a risk factor in the development of cervical cancer [90]. A combined phosphoproteome and transcriptome analysis of *C. trachomatis* infected cells demonstrated that *C. trachomatis* induced EMT was found to be mediated by ERK dependent phosphorylation of ETS2 repressor factor (ERF) and proto-oncogenic transcription factor ETS1 [54]. Inhibition of ERK activity via U0126 abrogated phosphorylation of these transcription factors and expression of their target genes in *C. trachomatis* infected cells. Thus, ERK signaling is important for regulation of transcription factors that control EMT.

*C. pneumoniae* has been associated with atherosclerosis and found in atherosclerotic lesions suggesting a causal role in atherogenesis [6,7]. Early studies implicated *C. pneumonaie-*induced signal transduction pathways in multiple cell types relevant to atherogenesis. Endothelial cells infected with *C. pneumonaie* exhibited increased phosphorylation of p44/p42 MAPK and increased activation/translocation nuclear factor–κB (NF-κB) [91]. Heat shock protein 60 (HSP60) also activated macrophages, endothelial cells, and smooth muscle cells resulting in induced expression of adhesion molecules and activation of NF-κB in addition to the production of proinflammatory cytokines [92,93]. Supporting evidence for these initial observations include studies that implicate *C. pneumoniae* in vascular smooth muscle cell (VSMC) proliferation. VSMC proliferation is responsible for intimal hyperplasia in early atherscelotic lesions. Specifically, *C. pneumoniae* and its HSP60 induces the proliferation of human VSMCs via Toll like receptor (TLR)4-mediated activation of p44/p42 (ERK1/2) kinase signaling [57]. The role of p44/p42 kinase signaling was also reported in *C. pneumoniae* induced proliferation of coronary artery smooth muscle cells [56]. Additionally, *C. pneumoniae* HSP60 induces the inflammatory response via activation of MAPK kinase 3 (MKK3)/p38 signaling enhancing lung inflammation [55]. Furthermore, MKK3 induces NF-κB activation by stimulating the nuclear kinase, mitogen- and stress-activated protein kinase (MSK) 1 initiating the inflammatory response [55].

## 8. Conclusions

Host kinases regulate host cell processes by phosphorylation of their target proteins. From entry and intracellular development to exit from the host cell, *Chlamydia* subverts many host kinase signaling pathways to ensure its survival. MEK/ERK and PI3K signaling pathways are among the most prominent kinase signaling networks utilized by *Chlamydia* in regulating entry, host cell apoptosis resistance, immune response, and even pathology associated with chlamydial infections. Several chlamydial effectors likely interact with but may also phosphorylated by host kinases during infection suggesting their activity may dependent on host signaling pathways. Elucidating the role of chlamydial effector phosphorylation is an intriguing topic for future studies.

## Figures and Tables

**Table 1:** Host kinases and their roles in chlamydial infection.

Host Kinase	Implicated Roles in Chlamydial Infection	References
Src, Abl, Syk	Phosphorylation T3SS effectors TarP and TepP. Important for entry, inclusion development and regulation of innate immune response	[17,18,19,20,21,22]
FAK	Recruited by TarP Actin remodeling during entry	[23,24]
SFKs (Src, Fyn)	Microdomain localization Microtubule dependent trafficking of inclusion to MTOC Intracellular development Lipid acquisition	[25,26,27]
MLCK	Microdomain localization with other elements of Myosin pathway Regulation of extrusion	[28,29,30]
RTKs (PDGFR, FGFR, EGFR, EPHA2)	Chlamydial entry and invasion F-actin assembly around inclusion Host cell survival	[18,31,32,33,34]
PI4KIIα	Generation of a pool of PI4P around inclusion Important for infectious progeny and inclusion formation	[35]
PI3K/AKT	Invasion Apoptosis resistance Chlamydial replication Dampening of innate immune response Sphingolipid acquisition Reduction of tumor suppressor protein p53	[21,34,36,37,38,39,40,41]
PDPK1	Apoptosis resistance	[42]
GSK3β	Interacts with Cpn1027 Recruitment by TepP	[21,43]
PKC	Resistance to apoptosis Microdomain localization Potential role in lipid acquisition	[44,45,46,47]
MEK/ERK	Lipid acquisition Bacterial replication Apoptosis resistance cytokine IL-8, IL-10 induction *Chlamydia* induced epithelial-to-mesenchymal transition Proliferation of vasculature smooth muscle cells	[18,23,31,32,36,40,48,49,50,51,52,53,54,55,56,57,58]
